# Follow-up study of anti-SARS-CoV-2 IgG antibody response in COVID-19 patients up to 6 months after infection

**DOI:** 10.2217/fmb-2022-0006

**Published:** 2022-08-04

**Authors:** Atalay Eray, Karamese Murat, Gumus Abdullah, Kahraman Ihsan, Kelesoglu Royca, Ozgur Didem, Tutuncu E Ediz

**Affiliations:** ^1^Department of Internal Medicine, Kafkas University, Kars, 36100, Turkey; ^2^Department of Medical Microbiology, Kafkas University, Kars, 36100, Turkey; ^3^Department of Infectious Diseases & Clinical Microbiology, Kafkas University, Kars, 36100, Turkey

**Keywords:** antibody response, COVID-19, follow-up study, SARS-CoV-2

## Abstract

**Introduction:** The aim of the current study was to investigate the relationship between lung involvement of SARS-CoV-2 and antibody levels of COVID-19 patients 3 and 6 months from the disease. **Methods:** A total of 156 participants were divided into two groups, Group 1: lung involvement (LI)-positive and Group 2: LI-negative. Biochemical parameters and anti-SARS-CoV-2 IgG antibody levels were measured. **Results:** The results showed that mean levels of urea, LDH, CRP, ferritin, neutrophil count and D-dimer were significantly higher in the LI-positive group. In addition, mean antibody levels in the 3rd and 6th months were higher in the LI-positive group (p < 0.005). **Discussion:** High antibody levels in LI-positive patients correlated with some immunologic and biochemical parameters. Further studies should be performed to determine protective antibody levels against reinfection, how long protective titers last and the mechanisms by which COVID-19 symptoms, demographics and comorbidities may drive higher antibody levels.

COVID-19, caused by SARS-CoV-2, was first declared in December 2019 in Wuhan, China, and has spread across the world. The disease is characterized by many typical symptoms such as dyspnea, high fever, cough, shortness of breath, pneumonia and loss of sense of smell/taste. There have been nearly 250 million confirmed infections and nearly 5 million deaths reported worldwide [1–3].

Lungs may be severely affected by SARS-CoV-2 and abnormalities reside mostly in the inferior lobes. The congestion in the lungs can be visible in lung CT scans; however, it is difficult to differentiate from interstitial pneumonia or other lung diseases and manual classification may be biased depending on the expert's opinion [4–6]. Chest CT imaging to evaluate pathological changes in the lung is a highly reliable, practical and rapid method for evaluating COVID-19 compared with other diagnostic tests (e.g., real-time PCR [RT-PCR]) [7]. In COVID-19 patients, disease severity is defined as follows: mild (no pneumonia on CT), moderate (pneumonia on CT but not requiring supplemental oxygen) and severe (requiring supplemental oxygen, ICU admission or mechanical ventilation) [8].

In the current literature, abnormal lung function has been reported in COVID-19 patients after discharge from the hospital; however, limited studies are available addressing long-term follow-up of COVID-19 patients. Additionally, studies are required to detect the level of anti-SARS-CoV-2 IgG antibody, because these antibody levels play a crucial role in understanding the immune response to aid in protection and recovery from repeated infections with SARS-CoV-2 [9–11]. Almost all countries have now begun to vaccinate people against SARS-CoV-2 infection. Although it is not known how long antibodies are protective, this issue has attracted close attention worldwide [12]. In this follow-up study, the investigators assess the relationship between lung involvement (LI) of the SARS-CoV-2 virus and antibody levels in COVID-19 patients 3 and 6 months after the disease.

## Methods

### Ethical approval

The current study was approved by both the Local Ethics Committee of Kafkas University Faculty of Medicine (approval date/number: 12.02.2021/2021-02-10516-23-51) and the Republic of Turkey Ministry of Health COVID-19 Scientific Research Evaluation Commission (approval date/number: 11.03.2021/2021/29). The authors assert that all procedures contributing to this work comply with the ethical standards of the relevant national and institutional committees on human experimentation and with the Helsinki Declaration of 1975, as revised in 2008. A written informed consent form was signed by each participant.

### Patient selection

A total of 156 participants were included in this study. The only inclusion criterion was having been diagnosed as SARS-CoV-2 by the RT-PCR technique. After the diagnosis of SARS-CoV-2, chest CT scans were performed and LI (i.e., peripherally, lower lobe predominant, multiple, bilateral ground glass opacities) was evaluated. At that point, patients were divided into two groups, Group 1: LI-positive and Group 2: LI-negative.

### Final study population

Blood samples were taken in the 3rd and 6th months after the diagnosis of COVID-19. A total of 48 patients whose blood samples could not be obtained 6 months after diagnosis were excluded from the study. The study was completed with 108 participants.

### Sample & data collection

Blood samples (~6–7 ml) were placed into blood tubes with EDTA and centrifuged at 3500 r.p.m. for 10 min. Serum samples were separated and stored at -80 °C until the study day. Biochemical parameters such as urea, creatinine, aspartate aminotransferase (AST), alanine aminotransferase (ALT), creatine kinase (CK), lactate dehydrogenase (LDH), C-reactive protein (CRP), ferritin, hemoglobin (HMG), neutrophil (NEU), lymphocyte (LYM), platelets (PLT), D-dimer and white blood cell (WBC) were measured by automated bioanalyzer machine. Data regarding age, gender, weight, height and comorbid diseases were obtained from the patients.

### Study steps

To detect antibody levels, the anti-SARS-CoV-2 QuantiVac ELISA (IgG) test kit (Catalogue number: EI-2606-9601-10-G, Euroimmun, Luebeck, Germany), which applies a recombinant S1 subunit of the SARS-CoV-2 spike protein enabling detection of IgG antibodies, was used. All kit contents and 96-well ELISA microplates were brought to room temperature before the study. The first well was a negative control, the second well was a positive control and 3–8 wells were calibrators (1, 10, 20, 40, 80 and 120 RU/ml, respectively). Diluted samples (100 μl; 1:101) were added to each sample well. After incubation (37 °C for 60 min) and washing with phosphate buffer saline (PBS), a 100-μl enzyme conjugate (peroxidase-labeled antihuman IgG) was added to each well. After further washing, 100 μl of chromogen/substrate solution was added and incubated at 37 °C for 30 min under darkened conditions. Finally, a 100-μl stop solution was added to each well and the plate was read at a wavelength of 450 nm by a Multiskan™ GO UV/Vis microplate spectrophotometer (Thermo Scientific, Schwerte, Germany) [13].

### Interpretation of the data

Results were evaluated by calculating a ratio of the optical density (OD) of the samples over the OD of the calibrators, ranging from 1–120 RU/ml. Quantitative results obtained in RU/ml were converted to international units (IU/ml) by multiplying by 3.2, in accordance with WHO specifications. If the ratio was under 25.6 IU/ml, it was considered negative; if it was between 25.6 and 35.2 IU/ml, it was considered borderline positive; and if it was above 35.2 IU/ml, it was considered positive.

### Statistical analysis

Data were analyzed using IBM SPSS version 21.0 statistical software (IBM, NY, USA). The number (n), percentage (%), mean, standard deviation (SD) and minimum and maximum values are given as descriptive statistics. The independent samples *t*-test or Mann–Whitney *U* test were used to compare numerical variables. All p-values were based on a two-sided test of statistical significance and significance was accepted at the level of p < 0.05.

## Results

### Demographic data

A total of 108 COVID-19 patients participated in this study. Descriptive data are presented in Table 1. The mean age was 44.79 ± 7.54 years in the LI-positive group and 39.27 ± 9.97 years in the LI-negative group. There were 48 male (76.2%) and 15 (23.8%) female patients in the LI-positive group and 20 male (44.4%) and 25 (55.6%) female patients in the LI-negative group. The clinical characteristics of the study participants are also reported in Table 1.

**Table 1.** Characteristics of COVID-19 patients.

Parameters	LI-positive group	LI-negative group	Z/X^2^	p-value
Mean age	44.79 ± 7.54 years	39.27 ± 9.97 years	-2.878	**0.004**
Gender	48 male (76.2%) 15 female (23.8%)	20 male (44.4%) 25 female (55.6%)	11.345	**0.001**
Mean weight	87.51 ± 17.57 kg	74.51 ± 15.14 kg	-3.530	**0.000**
Mean height	172.41 ± 8.50 cm	167.98 ± 6.39 cm	-2.667	**0.008**
Mean urea level	32.58 ± 13.14 mg/dl	21.92 ± 13.40 mg/dl	-2.636	**0.008**
Mean creatinine level	1.01 ± 0.036 mg/dl	0.87 ± 0.021 mg/dl	-1.493	0.135
Mean AST level	36.95 ± 29.35 U/l	23.30 ± 9.01 U/l	-2.087	0.337
Mean ALT level	33.95 ± 26.44 U/l	28.89 ± 18.66 U/l	-0.958	0.338
Mean CK level	335.75 ± 95.59 U/l	240.59 ± 96.83 U/l	-0.607	0.994
Mean LDH level	331.78 ± 83.91 U/l	221.89 ± 41.36 U/l	-3.002	**0.003**
Mean CRP level	3.10 ± 3.46 mg/l	0.95 ± 1.17 mg/l	-3.228	**0.001**
Mean ferritin level	419.2 ± 428.4 ng/ml	183.20 ± 162.72 ng/ml	-2.654	**0.008**
Mean HMG level	14.97 ± 1.44 g/dl	14.56 ± 2.56 g/dl	-0.540	0.589
Mean NEU level	3.61 ± 1.65 10^3^/mm^3^	2.69 ± 0.97 103/mm^3^	-2.215	**0.027**
Mean LYM level	1.57 ± 0.57 10^3^/mm^3^	2.69 ± 0.97 103/mm^3^	-0.282	0.778
Mean PLT level	210.88 ± 59.42 10^3^/mm^3^	205.56 ± 46.24 103/mm^3^	-0.279	0.780
Mean D-dimer level	816.13 ± 125.41 μg/ml	403.23 ± 443.90 μg/ml	-2.268	**0.023**
Mean WBC level	5.76 ± 1.98 10^3^/mm^3^	4.36 ± 1.75 103/mm^3^	-2.127	**0.033**
Mean antibody level (3 months)	293.58 ± 206.43 IU/ml	196.28 ± 121.54 IU/ml	-2.125	**0.034**
Mean antibody level (6 months)	203.64 ± 167.86 IU/ml	79.28 ± 89.91 IU/ml	-3.999	**0.000**

The bold text indicates a statistically significant difference.

ALT: Alanine aminotransferease; AST: Aspartate transaminase; CK: Creatine kinase; CRP: C-Reactive protein; HMG: Hemoglobin; LDH: Lactate dehydrogenase; LYM: Lymphocyte; NEU: Neutrophil; PLT: Platelet; WBC: White blood cell.

### Levels of anti-SARS-CoV-2 IgG antibody & biochemical parameters

The results showed that urea (32.58 ± 13.14 mg/dl), LDH (331.78 ± 83.91 U/l), CRP (3.10 ± 3.46 mg/l), ferritin (419.2 ± 428.4 ng/ml), neutrophil (3.61 ± 1.65 103/mm^3^) count and D-dimer (816.13 ± 125.41 μg/ml) values were higher in the LI-positive group than in the LI-negative group and was a statistically significant difference (p < 0.05). There were no significant between-group differences in AST (36.95 ± 29.35 U/l), ALT (33.95 ± 26.44 U/l), CK (335.75 ± 95.59 U/l), HMG (14.97 ± 1.44 g/dl), LYM (1.57 ± 0.57 103/mm^3^) or PLT (210.88 ± 59.42 103/mm^3;^ p > 0.05). In addition, mean antibody levels at 3 and 6 months were higher in the LI-positive group (p < 0.05). On the other hand, smoking status and having at least one comorbid disease were evaluated and no statistically significant relationships were detected between those parameters and mean antibody levels (Table 2). Additionally, some patients had antibody levels under 25.6 IU/ml (negative) at both 3 and 6 months, as seen in Table 3.

**Table 2.** Smoking status and comorbid diseases.

Parameters	LI-positive group	LI-negative group
Smoking – Yes – No	5 (7.9%) 58 (92.1%)	12 (26.7%) 33 (73.3%)
Comorbid diseases – None – COPD – Asthma – HT – DM – Others	34 (54.0%) 3 (4.8%) 2 (3.2%) 8 (12.7%) 5 (7.9%) 11 (17.5%)	23 (51.1%) 3 (6.7%) 4 (8.9%) 4 (8.9%) 3 (6.7%) 8 (17.8%)

	**Z**	**p-value**
Antibody level X smoking	-0.628	0.530
Antibody level X comorbid diseases	-1.951	0.051

COPD: Chronic obstructive pulmonary disease; LI: Lung involvement.

**Table 3.** Data for patients with <25.6 IU/ml antibody levels.

Parameters	LI-positive		LI-negative	
	3 months	6 months	3 months	6 months
Total patient no.	86	63	70	45
Patients with <25.6 IU/ml antibody level (negative)	1	6	7	11
%	1.16	9.52	10.00	24.44

LI: Lung involvement.

## Discussion

In the COVID-19 pandemic, early diagnosis is necessary to both improve prognosis and reduce the spread of the virus. According to guidelines, patients with suspected COVID-19 should be tested using RT-PCR as the gold standard to detect the viral RNA in respiratory tract samples by using suitable PCR primers and then, if necessary, chest imaging [14].

Xu *et al.* [15] reported pathological pulmonary manifestations in COVID-19 patients, including bilateral alveolar damage with cellular fibromyxoid exudates, exfoliation of pneumocytes, edema and hyaline membrane formation. Luo *et al.* in their study on 187 cases found that patients with COVID-19 positive pulmonary findings had advanced age; higher fever; higher neutrophil, CRP, ESR and LDH levels; and there was a positive correlation between the level of systemic inflammation and radiographic features. The mean age of the LI-positive group was also higher than the LI-negative group [16]. Similarly, in the current study, we found that patients with positive lung findings had higher inflammatory parameters than patients with negative lung findings, and the mean age was higher in the LI-positive group.

Antibodies are important components in the formation of protective immunity against new viral infections such as SARS-CoV-2 [17]. Many studies report that IgG antibodies against SARS-CoV-2 spike and receptor binding domain (RBD) antigens are detected in the blood of >90% of patients 10–11 days after the onset of symptoms [18–20]. One study reported that 99.33% (298/300) were detected as positive in terms of S-RBD SARS-CoV-2 antibodies [21]. The antibody response to SARS-CoV-2 is typical, with virus-specific IgM peaking in the acute phase about 2–5 weeks after the onset of illness and falling after 3–5 weeks. In most cases, IgG then peaks (3–7 weeks after disease onset) and then flattens out and persists for at least 8 weeks [17,22]. However, whether SARS-CoV-2 antigen-specific IgG levels persist remains debated. Coppeta *et al.* performed a study on 793 healthcare workers who had two doses of mRNA vaccine and reported that S-RBD antibodies persist for up to 250 days after the second dose of vaccine [23]. Additionally, Isho *et al.* confirmed that serum and salivary IgG antibodies to SARS-CoV-2 are maintained for at least 3 months from the onset of symptoms in most COVID-19 patients [5]. In the current study, antibody levels measured 3 and 6 months after the onset of symptoms were found to be preserved in the serum, and the 3-month antibody level was higher than the 6-month antibody level.

Carsetti *et al.* [24] in their studies investigating the relationship between COVID-19 clinic and antibody level, stated that monocytes produced relatively late during infection and high IgG levels characterize the severe course, while a mild increase in monocytes and rapidly decreasing antibody levels characterize the mild course. In a similar study by Amjadi *et al.* [25], 113 blood samples in the 5th week and 79 blood samples in the 3rd month were collected from COVID-19 patients to determine the level of anti-SARS-CoV-2 IgG antibody. They reported that greater disease severity, older age, male sex, higher BMI and higher Charlson Comorbidity Index score consistently correlate with higher antibody titers. Garcia-Beltran *et al.* [26] examined antibody responses in 113 COVID-19 patients and found that severe cases resulting in intubation or death exhibited increased inflammatory markers, lymphopenia, proinflammatory cytokines and high RBD antibody levels. Another cohort study [27] performed on 140 confirmed COVID-19 patients, including patients with mild symptoms and more severe forms, reported high correlations between antibody levels, comorbidity and disease severity. On the other hand, a study from Turkey performed on 72 patients with laboratory-confirmed COVID-19 reported that the prognosis and severity of chest CT involvement in COVID-19 are related to levels of immune cells. They recommended that early evaluation of CT scores and immune parameters in COVID-19 patients is essential in determining severe patients [28]. Similarly, Lucas *et al.* [29] analyzed humoral immune responses in 229 patients with asymptomatic, mild, moderate and severe COVID-19 over time to probe the nature of antibody responses in disease severity and mortality. They observed a correlation between IgG levels, length of hospitalization and clinical parameters associated with worse clinical progression.

The results of these studies support the main hypothesis of the current work. We also believe there should be a correlation between the level of IgG antibodies and clinical features such as LI and some blood parameters. In the current study, antibody levels were higher in the LI-positive group than in the LI-negative group. The mean weight and height, mean urea level, mean LDL level, mean CRP level, mean ferritin level, mean NEU level, mean D-dimer level and mean WBC level were statistically significant in LI-positive patients compared with LI-negative patients. In addition, Elsande *et al.* [19] reported that in 236 PCR-positive SARS-CoV-2-infected patients, antibody negativity was 22.2% versus 2.6% after mild and severe disease, which was similar to this study. Chia *et al.* performed a cohort study on 517 patients who had COVID-19 disease. Blood samples were collected on days 60, 90 and 180 from 164 patients who had mild, moderate or severe LI. The results showed an association between neutralizing antibodies and severe COVID-19 clinical symptoms such as pneumonia (Table 3) [8].

This study aimed to investigate the relationship between LI and antibody levels in patients with SARS-CoV2 detected by RT-PCR. In the 3rd month after COVID-19 disease, the mean antibody level in LI-positive patients was higher than the level in LI-negative patients (Z = -2.125; p = 0.034). In the 6th month after COVID-19 disease, the mean antibody level in LI-positive patients was also higher than the level in LI-negative patients (Z = -3.999; p = 0.000).

## Conclusion

The data describing COVID-19 and its outcomes are limited. The authors aimed to contribute to the current literature in terms of the relationship between anti-SARS-CoV-2 IgG antibody levels and clinical features, including radiologic, immunologic and biochemical parameters. The prevalence and prognosis of SARS-CoV-2 pneumonia vary considerably among individuals. High antibody levels in LI-positive patients correlated with some immunologic and biochemical parameters. Further studies should be performed to determine protective antibody levels against reinfection, how long protective titers last and the mechanisms by which COVID-19 symptoms, demographics and comorbidities may drive higher antibody levels.

**Summary points**

- According to the current literature, abnormal lung function has been detected in COVID-19 patients after being discharged from the hospital; however, limited studies are available that describe the long-term follow-up of COVID-19 patients.
- Antibody response after exposure to the SARS-CoV-2 virus has been shown to decrease over time; however, there is limited data about anti-SARS-CoV-2 IgG antibody levels at 3 and 6 months postinfection.
- This study was designed to investigate the relationship between lung involvement (LI) and antibody levels in COVID-19 patients. The mean antibody level in LI-positive patients was higher than the level in LI-negative patients in both 3rd- and 6th-month blood samples.
- Further studies should be performed to determine protective antibody levels against reinfection, how long protective titers last and the mechanisms by which COVID-19 symptoms, demographics and comorbidities may drive higher antibody levels.
